# Optical monitoring of glutamate release at multiple synapses in situ detects changes following LTP induction

**DOI:** 10.1186/s13041-020-00572-x

**Published:** 2020-03-13

**Authors:** Olga Kopach, Kaiyu Zheng, Dmitri A. Rusakov

**Affiliations:** Queen Square Institute of Neurology, University College London, Queen Square, London, WC1N 3BG UK

**Keywords:** Glutamate release, Optical glutamate sensor, LTP, Two-photon excitation imaging, Acute hippocampal slices

## Abstract

Information processing and memory formation in the brain relies on release of the main excitatory neurotransmitter glutamate from presynaptic axonal specialisations. The classical Hebbian paradigm of synaptic memory, long-term potentiation (LTP) of transmission, has been widely associated with an increase in the postsynaptic receptor current. Whether and to what degree LTP induction also enhances presynaptic glutamate release has been the subject of debate. Here, we took advantage of the recently developed genetically encoded optical sensors of glutamate (iGluSnFR) to monitor its release at CA3-CA1 synapses in acute hippocampal slices, before and after the induction of LTP. We attempted to trace release events at multiple synapses simultaneously, by using two-photon excitation imaging in fast frame-scanning mode. We thus detected a significant increase in the average iGluSnFR signal during potentiation, which lasted for up to 90 min. This increase may reflect an increased amount of released glutamate or, alternatively, reduced glutamate binding to high-affinity glutamate transporters that compete with iGluSnFR.

## Introduction

Hebbian postulates, which rationalise the principles of memory formation in the brain [1], have found their first experimental verification in the long-term potentiation (LTP) of excitatory transmission [2, 3]. The majority of excitatory synapses in the cortex operate by releasing glutamate from presynaptic axons, the process that underpins rapid information processing and storage by neural circuits. Following decades of debate, it has been argued that the prevailing cellular mechanism underlying LTP rests with an increased current through postsynaptic receptors [4]. Experimental evidence for the alternative hypothesis, such as an increase in glutamate release probability [5–7], has been countered by an elegant hypothesis of silent synapses [8, 9] and by documenting no increases in astroglial glutamate uptake post-induction [10, 11]. However, the LTP-associated boost of release probability at non-silent synapses has subsequently been reported [12, 13] whereas no change in overall glutamate release can reflect hetero-synaptic depression at non-active connections [14] or, more generally, rapid (pre) synaptic scaling [15, 16]. The uncertainty has remained, largely because documenting glutamate release at individual synapses has had to rely on its physiological consequences rather than on release readout per se.

The advent of FM dyes decades ago was an important step in providing optical tools to detect exocytosis of synaptic vesicles [17, 18]. More recently, the emergence of genetically encoded optical sensors for glutamate [19] has finally enabled direct monitoring of its release at individual synaptic connections. We showed earlier that, in certain imaging conditions, fluorescent glutamate ‘sniffers’ of the iGluSnFR family provide robust readout of glutamate release at identified synapses in organised brain tissue [20, 21], including in vivo [22]. In the present study, we take advantage of this approach in an attempt to understand changes in glutamate release properties at hippocampal Schaffer collateral axons, under the classical protocol of LTP induced by high-frequency afferent stimulation. We monitor LTP induction in the bulk of tissue, and analyse optical glutamate signals in arbitrary samples of presynaptic axonal boutons, which may correspond to both potentiated and non-potentiated synapses. In these settings, we aim to assess changes in glutamate release at individual synapses, and in the bulk of synaptic population.

## Results

### Viral delivery of iGluSnFR in neonates for multi-synapse glutamate imaging in situ

**Fig. 1:**
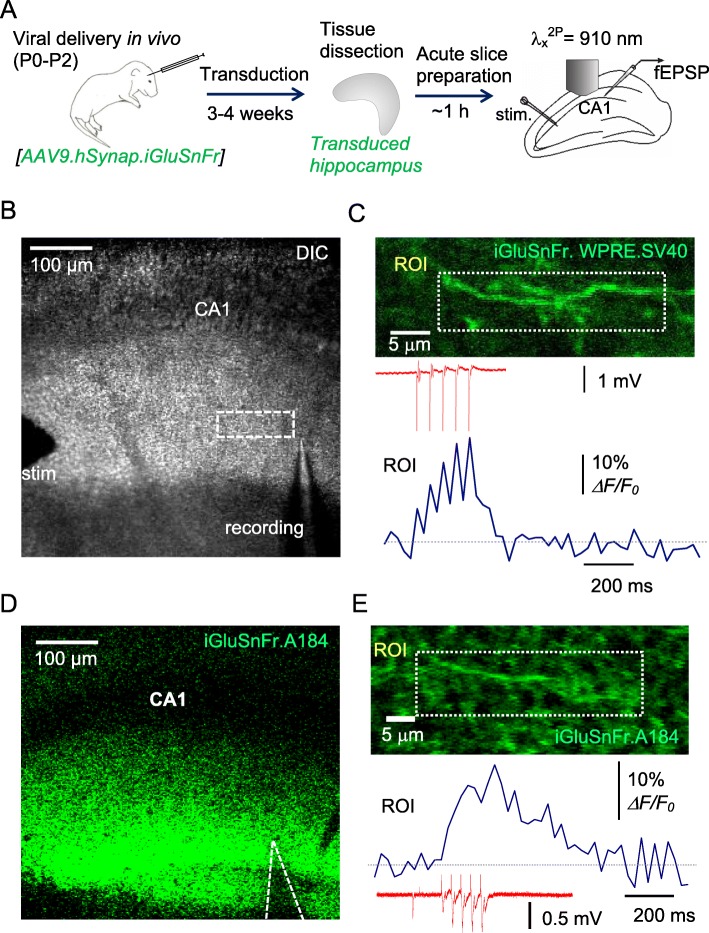
Monitoring glutamate release from multiple axons ex vivo in hippocampal slices labelled with iGluSnFR through viral transduction in vivo. **a** A diagram depicting viral ICV injections in neonates (P0-P2) followed by AAV transduction (3–4 weeks), dissection of hippocampi, and acute slice preparation for two-photon excitation imaging coupled with electrophysiology (Schaffer collateral stimulation and fEPSP recording in *S. radiatum*). **b** Experimental arrangement as seen in the microscope (DIC channel); stimulating and recording electrodes are seen; dotted rectangle, ROI for imaging. **c** Image, axon fragment in *S. radiatum* (ROI as in B) as seen in the green channel (AAV9.hSynap.iGluSnFR.WPRE.SV40 fluorescence; 50-frame average). Upper trace, fEPSP response to afferent stimuli (five at 20 Hz, one-trial example); lower trace, the corresponding ROI-averaged *ΔF/F*_*0*_ signal time course (one-trial example). **d** Arrangement as in (**b**) but for the ‘slow-decay’ sensor variant AAV2/1.hSyn.SF.iGluSnFR.A184S (green channel shown). **e** Experiment as in (**c**) but for AAV2/1.hSyn.SF.iGluSnFR.A184S; notation as in (**c**)

In our previous studies, we introduced optical glutamate sensors in the hippocampal neuropil via stereotaxic viral delivery in young animals [20] or via biolistic transfection in organotypic brain slices [21, 23]. However, brain injections in adults face challenges, such as potential interference with the tissue designated for acute slices, whereas the functional morphology of organotypic slices might not fully represent that of intact tissue. We, therefore, sought to explore viral transduction in vivo via neonatal intracerebroventricular (ICV) injections (Fig. 1a), aiming at efficient transgene expression in neurons, for up to 6 weeks post-infection for subsequent ex vivo imaging.

We employed the new generation of AAV-based sensor variant with a relatively high off-rate, AAV9.hSynap.iGluSnFR.WPRE.SV40, but also used the recently described low off-rate sensor variant, SF-iGluSnFR.A184S [24] for comparison. Although AAV9 appeared to penetrate more readily after ICV administration [25] than did AAV2/1, at three to 4 weeks post-injection, both methods provided efficient labelling of Schaffer collateral fragments in *S. radiatum* (Fig. 1c-e). The robust level of expression was maintained for at least 6 weeks post-infection, which made it suitable for ex vivo experiments in acute slices from young adult animals.

To validate the method, we set out to monitor iGluSnFR fluorescence intensity integrated across the region of interest (ROI, the area incorporating several axonal boutons) during electric stimulation of Schaffer collaterals (five stimuli 50 ms apart; imaging settings as described earlier [20]). For time-lapse imaging, we employed frame-scanning mode providing rapid sampling rate (pixel dwell time 0.5 μs, frame time ~ 25 ms) across the area of interest (256 × 96 pixels, Fig. 1c). The recorded data sets were arranged as T-stacks, consisting of multiple frame scans (typically 35 to 50, depending on the duration of recording). We thus achieved reliable imaging of the dynamics of glutamate release across the sampled tissue fragment (using a galvo mirror scanhead), with clear separation of five responses to individual electric stimuli (Fig. 1c; fEPSP and *ΔF/F*_*0*_ signal traces; one-trial example). Our attempts to achieve a faster frame rate using a continuous resonant-scanner mode (with a Femtonics Femto-SMART scope) could not obtain a suitable trade-off between laser power and pixel dwell time to generate satisfactory signals without tissue damage, at least under the current protocol. Specific (non-continuous) regimes for resonant-scanner imaging may be required to achieve that.

A similar experiment using the slow-unbinding A184S sensor variant (Fig. 1d) revealed robust stimulus-evoked rises in the iGluSnFR intensity (Fig. 1e). However, this sensor variant did not seem to provide reliable separation between individual responses to five stimuli applied at 20 Hz (Fig. 1e; *ΔF/F*_*0*_ trace, one-trial example), thus pointing to the corresponding limitations in temporal resolution.

### Multi-synapse imaging of glutamate release at individual axonal boutons

**Fig. 2:**
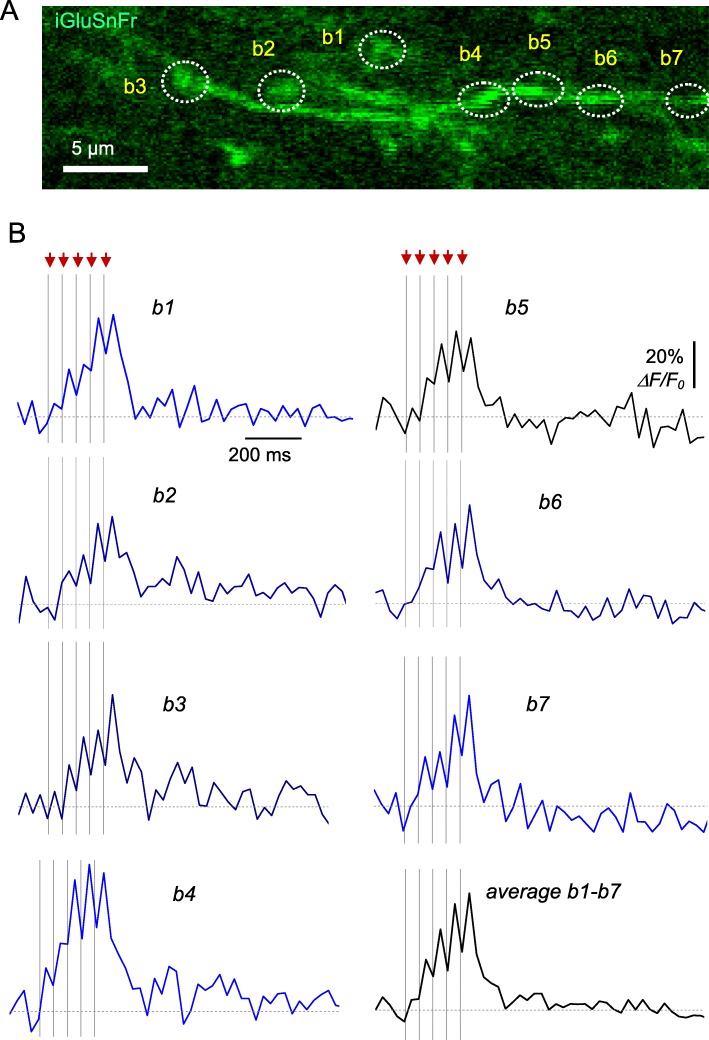
Optical monitoring of glutamate release from multiple synapses using the AAV9.hSynap.iGluSnFR.WPRE.SV40 imaging in fast frame-scan mode. **a** Axonal fragments in *S. radiatum* (same region as in Fig. 1c), showing candidate presynaptic boutons (b1-b8). Image is average of 50 frames of the T-stack. **b** Traces, *ΔF/F*_*0*_ signal time course within individual ROIs that correspond to boutons b1-b7 shown in (**a**) and the b1-b7 average trace, as indicated, during afferent stimulation (five pulses at 20 Hz; four-trial average)

**Fig. 3:**
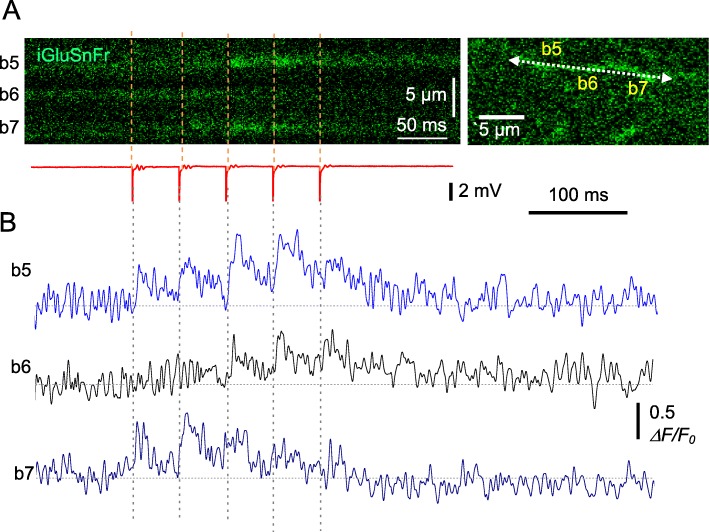
Documenting glutamate release from multiple axonal boutons using linescan imaging mode. **a** Linescan image (left) depicting the iGluSnFR.WPRE.SV40 fluorescence time course in three axonal boutons (b5-b7, right; ROI as in Fig. 2) during afferent stimulation (five pulses at 20 Hz), with fEPSP monitoring (red trace). **b** Traces, *ΔF/F*_0_ signal time course recorded as shown in (**a**) (one-trial example).

We next asked if the chosen frame-scanning method is sufficiently sensitive to document glutamate release at individual axonal boutons. We therefore used the recorded image-frame stacks to analyse fluorescence dynamics at small ROIs associated with individual axonal boutons (Fig. 2a). The fluorescence dynamics at individual selected boutons showed that recording sensitivity and signal-noise ratios were sufficient, in principle, to document individual glutamate releases (Fig. 2b; *ΔF/F*_0_ traces, four-trial average), at least in baseline conditions. For comparison purposes, we recorded a fragment of the same axon (as Fig. 2a) in linescan mode, which provides high temporal resolution (~ 1.45 ms). The fluorescence dynamics thus recorded from three boutons of interest (Fig. 3a, bouton numbers as in Fig. 2a; one-trial example) was qualitatively similar to that obtained in the frame-scanning mode (compare boutons 5–7 in Fig. 2b and Fig. 3b).

### Imaging glutamate release during LTP induction

One of the main advantages of the frame-scan mode (with galvo mirrors), as opposed to various linescan options, is relatively low overall (cumulative) laser exposure per pixel yet sufficient pixel dwell time to generate enough photons. Firstly, this lowers the propensity for irreversible photo-damage that may occur in cellular structures under intense laser light. Secondly, it reduces photobleaching of the fluorescent indicator, which has been a key prerequisite for stable longer-term imaging. As pointed out above, available parameters of the continuous resonant scanning fell outside the optimal range for the present protocol.

**Fig. 4:**
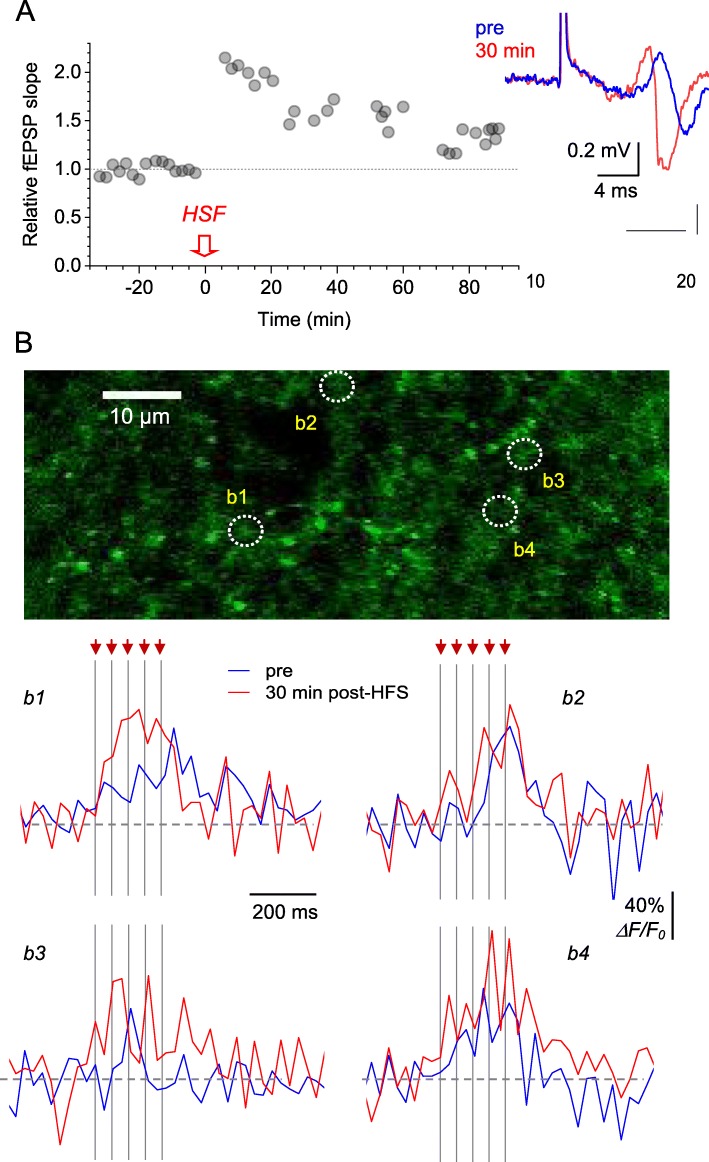
Optical glutamate signal at individual axonal boutons during LTP induction. **a** Characteristic time course of the fEPSP slope recorded in *S. radiatum* following LTP induction by high frequency stimulation (HFS, one-slice example). Traces, the corresponding fEPSP examples in baseline conditions (blue) and 30 min after LTP induction (red). **b** Image, ROI in *S. radiatum* (iGluSnFR.WPRE.SV40 channel) showing 4 axonal boutons, b1-b4, designated for glutamate release monitoring. Traces, iGluSnFR *ΔF/F*_0_ signal recorded from boutons b1-b4 before (blue) and ~ 30 min after (red) LTP induction. Traces are single-trial examples; arrows and dotted lines, afferent stimulus timestamps

We therefore set out to explore our imaging method in an attempt to document changes, if any, of glutamate release during the high-frequency stimulation (HFS)-induced LTP. The classical LTP induction protocol in iGluSnFR-expressing acute slices produced a reliable increase in the fEPSP slope, lasting for up to 90 min post-induction (example in Fig. 4a). In selected areas of *S. radiatum*, we thus identified groups of candidate axonal boutons that responded to afferent stimulation but also remained firmly in focus during the experiment, to reduce any bias associated with focal drift (Fig. 4b). The boutons selected based on this mandatory criterion, were not necessarily the boutons showing the best signal-to-noise ratios of their *ΔF/F*_*0*_ responses (this may also relate to varied iGluSnFR expression). Whilst individual boutons displayed varied effects of LTP induction on the fluorescence dynamics of iGluSnFR, they nonetheless appeared to indicate a clear trend towards an increase in the *ΔF/F*_*0*_ signal amplitude (example in Fig. 4c).

**Fig. 5:**
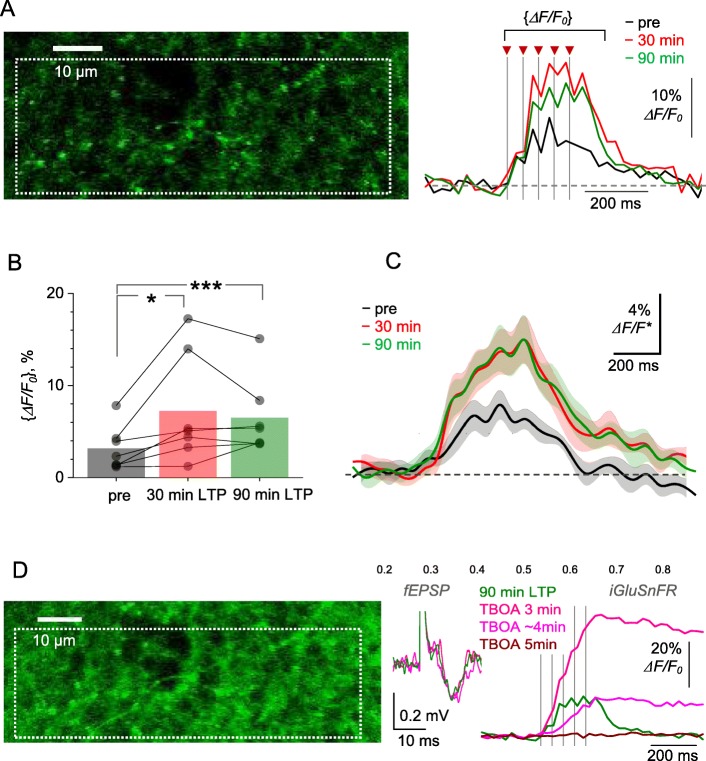
LTP induction at CA3-CA1 synapses boosts optical glutamate signal in the *S. radiatum* neuropil. **a** Image, axon fragment in *S. radiatum* showing the area with multiple axonal boutons (dotted rectangle, iGluSnFR.WPRE.SV40 channel) for the analysis of average iGluSnFR *ΔF/F*_0_ signal (right traces), as shown before (pre), ~ 30 min after (red), and 90 min after HFS. One-slice example; traces, singletrial examples; arrows and dotted lines, afferent stimulus timestamps. Averaging interval for calculating {*ΔF/F*_0_} values is shown. **b** ROI-average iGluSnFR {*ΔF/F*_0_} values in baseline conditions (pre), and at 30 min and 90 min after LTP induction, as indicated. Connected dots, individual slice data; bars, average values (*n* = 7). *p < 0.04; ***p < 0.005. **c** Average iGluSnFR *ΔF/F*_*0*_ signal traces (line ± shaded area, mean ± SEM, n = 7) normalised to their {*ΔF/F*_0_} value in baseline conditions, in each individual preparation, and rescaled to illustrate the ‘average *ΔF/F*_0_ traces’ across preparations (ΔF/F*). **d** Experiment as in (**a**) but following the blockade of glutamate transporters with 50 μM TBOA, at 90 min after LTP induction. fEPSP and iGluSnFR traces illustrate single trials recorded at different time points after TBOA application onset, as indicated; one-slice example, notations as in (**a**). Note that no *ΔF/F*_*0*_ signal (red) may reflect saturation of the baseline fluorescence *F*_*0*_

This trend was more prominent when the area-integrated *ΔF/F*_*0*_ signals (as in Fig. 1c, e) were compared (Fig. 5a). To evaluate this quantitatively, we first measured the iGluSnFR signal amplitude {*ΔF/F*_*0*_}, the mean *ΔF/F*_*0*_ value measured over 300 ms after the first stimulus onset (Fig. 5a, traces), 1–5 min prior to LTP induction, and 30 min and 90 min after LTP induction. Comparing these {*ΔF/F*_*0*_} values within individual slices revealed a significant increase after LTP induction (from 3.1 ± 0.9% to 7.2 ± 2.2% at 30 min post-HFS, *p* < 0.035; to 6.5 ± 1.6% at 90 min post-HFS, *p* < 0.005; *n* = 7 slices, paired *t*-test). Second, we compared full *ΔF/F* responses at the same time points. To achieve paired comparison, we normalised post-HFS traces by the {*ΔF/F*_*0*_} value of the pre-LTP response, within each individual preparation (slice), and then re-scaled all the traces to match the sample-average {*ΔF/F*_*0*_} value in baseline conditions (Fig. 5c). Again, this paired-comparison design revealed a prominent increase in the *ΔF/F*_*0*_ signal at 30 and 90 min after LTP induction (Fig. 5c). Whether such an increase necessarily indicates a greater amount of evoked glutamate release is discussed below.

### Blocking astroglial glutamate transport saturates iGluSnFR signal

Because the *ΔF/F*_*0*_ signal we record reflects glutamate binding to iGluSnFR molecules, it may compete with other (invisible) binding sites for glutamate in the neuropil. It has been well established that, once released from presynaptic boutons in the hippocampus, > 90% of glutamate molecules are bound and taken up by high-affinity astroglial glutamate transporters [26]. These transporters will therefore compete with iGluSnFR for glutamate binding and removal from the extracellular space, prompting a hypothesis that their inhibition could boost the iGluSnFR signal. To test this, we added the transporter blocker TFB-TBOA to the bath (50 μM), after recording a reliable *ΔF/F*_*0*_ response 90 min after LTP induction. Within 3 min after TBOA application, afferent stimulation induced a large, virtually irreversible increase in the iGluSnFR *ΔF/F*_*0*_ signal (Fig. 5d). The signal has become undetectable within the next few minutes, most likely due to the progressive saturation of iGluSnFR by the excess of extracellular glutamate in TBOA (Fig. 5d). At the same time, TBOA had little effect on the fast fEPSPs (Fig. 5d, fEPSP traces), reflecting no detectable influence on glutamate release, in line with earlier reports [27, 28]. These results indicate that the LTP-associated increase in the iGluSnFR *ΔF/F*_*0*_ signal could potentially be related to the reduced presence of astroglial glutamate transporters in the perisynaptic environment.

## Discussion

In this study, we took advantage of the recently developed, genetically encoded optical iGluSnFR sensors for glutamate [19, 24], in an attempt to detect changes in glutamate release following the induction of LTP. We have successfully transduced the sensors in hippocampal Schaffer collateral fibres using neonatal viral infection. We explored the suitability of the fast frame-scanning (two-photon excitation) imaging mode for monitoring optical glutamate signals from multiple axonal boutons, thus identifying some advantages and limitations, in the context. The key advantage rests with the reduced exposure to laser light and the ability to record from multiple synaptic connections, in some cases with satisfactory sensitivity and temporal resolution. This is, however, somewhat offset by the fact that during longer-term recordings and / or intense electric stimulation the tissue is likely to drift while also altering its morphological features, albeit on the microscopic scale. Because such movements could alter geometry and position of the fluorescence source(s), this could potentially bias the readout of dynamic optical recordings. Selecting the objects of interest, such as axonal boutons, based on their morphological stability, potentially leads to suboptimal sampling in terms of the signal-to-noise ratio. The future improvements of the technique could combine a better controlled labelling of axons, aiming at a sufficiently high level of iGluSnFR expression within targeted sub-microscopic structures. Improved out-of-focus detection could be achieved by using the vertically extended point-spread function of the two-photon excitation system [29], which is sometimes termed ‘Bessel beam’. The latter should help overcome the effects of 3D drift, and therefore improve the sampling procedure. Notwithstanding its potential limitations, another advantage of the present approach is its unbiased way of sampling axonal boutons. As the majority of excitatory synapses, or at least a significant proportion of them, are of low release probability [21, 30], one would expect boutons that are sampled in an unbiased way to show a relatively low glutamate signal on average, as we find here. It is likely that in other data sets, in which boutons are selected based on a high signal to noise ratio, represent higher release probability synapses.

The present approach has identified a significant increase in the average optical glutamate signal in the Schaffer collateral neuropil, up to ~ 90 min after LTP induction at CA3-CA1 synapses. This increase is unlikely to reflect the ‘transient-LTP’ component, which is expressed presynaptically but decays within 70–80 afferent discharges [31], or within 15–20 min under the present protocol. At first glance, the increase in iGluSnFR fluorescence must indicate an increased amount of released glutamate in response to afferent stimulation. However, the fluorescent signal of iGluSnFR reports glutamate molecules bound to the indicator. Any endogenous high-affinity glutamate buffer that competes with this binding process can potentially affect optical readout. Intriguingly, hippocampal neuropil is equipped with such a buffer, in the shape of high-affinity glutamate transporters that are expressed, at high density, on the surface of astroglia [26, 32, 33]. Thus, a significant decrease in the numbers of locally available glutamate transporters could boost glutamate binding to its optical sensor upon evoked release. Indeed, when we blocked astroglial glutamate transporters with TBOA, the iGluSnFR signal was first boosted then entirely saturated, reflecting an excess of extracellular glutamate. At the same time, no increase in glutamate release efficacy could be detected. Whether a similar mechanism is enacted during LTP induction is an intriguing and important question yet to be fully addressed.

On a more general note, the remaining uncertainty about release probability changes during LTP is unlikely to be resolved unambiguously without considering the effects of LTP induction on both potentiated and (neighbouring) non-potentiated synapses, and possibly on the local astroglial microenvironment. Similarly, it would seem important to employ an unbiased sampling method that would include all activated synapses (e.g., represented by axonal boutons or dendritic spines), regardless of their baseline synaptic efficacy or signal detectability.

## Methods

### Viral transduction for labelling axonal boutons within CA3-CA1 region

All animal procedures were conducted in accordance with the European Commission Directive (86/609/ EEC), the United Kingdom Home Office (Scientific Procedures) Act (1986). For the experiments, both male and female C57BL/6 J mice (Charles River Laboratories) were used. For ex vivo imaging of individual boutons, an AAV virus expressing the neuronal optical glutamate sensor, AAV9.hSynap.iGluSnFR.WPRE.SV40, supplied by Penn Vector Core (PA, USA) was injected into the cerebral ventricles of neonates. For viral gene delivery, pups, male and female (P0-P1), were prepared for aseptic surgery. To ensure proper delivery, intracerebroventricular (ICV) injections were carried out after a sufficient visualization of the targeted area [34]. Viral particles were injected in a volume 2 μl/hemisphere (totalling 5 × 10^9^ genomic copies), using a glass Hamilton microsyringe at a rate not exceeding of 0.2 μl/s, 2 mm deep, perpendicular to the skull surface, guided to a location approximately 0.25 mm lateral to the sagittal suture and 0.50–0.75 mm rostral to the neonatal coronary suture. Once delivery was completed, the microsyringe was left in place for 20–30 s before being retracted. Pups (while away from mothers) were continuously maintained in a warm environment to eliminate risk of hypothermia in neonates. After animals received AAV injections, they were returned to the mother in their home cage. Pups were systematically kept as a group of litters. Every animal was closely monitored for signs of hypothermia following the procedure and for days thereafter, to ensure that no detrimental side effects appear. For transduction of glutamate sensors in vivo, there were three- to four- weeks to suffice.

### Preparation of acute hippocampal slices

Acute hippocampal slices (350 μm thick) were prepared from three- to 4 week-old mice. The hippocampal tissue was sliced in an ice-cold slicing solution containing (in mM): 64 NaCl, 2.5 KCl, 1.25 NaH_2_PO_4_, 0.5 CaCl_2_, 7 MgCl_2_, 25 NaHCO_3_, 10 D-glucose and 120 sucrose, saturated with 95% O_2_ and 5% CO_2_. Acute slices were then transferred into a bicarbonate-buffered Ringer solution containing (in mM) 126 NaCl, 3 KCl, 1.25 NaH_2_PO_4_, 2 MgSO_4_, 2 CaCl_2_, 26 NaHCO_3_, 10 D-glucose continuously bubbled with 95% O_2_ and 5% CO_2_ (pH 7.4; 300–310 mOsmol). Slices were allowed to rest for at least 60 min before the recordings started. For recordings, slices were transferred to a recording chamber mounted on the stage of an Olympus BX51WI upright microscope (Olympus, Tokyo, Japan) and superfused at 31–33 °C.

### Two-photon (2P) excitation fluorescent imaging of glutamate release

2P excitation microscopy was carried out using an Olympus FV10MP imaging system optically linked to a Ti:Sapphire MaiTai femtosecond-pulse laser (SpectraPhysics-Newport), equipped with galvo scanners, and integrated with patch-clamp electrophysiology. Acute hippocampal slices were illuminated at λ_x_^2P^ = 910 nm (iGluSnFR optimum) in the green emission channel. In *s.radiatum* of area CA1, we focused on axonal fragments that (a) were expressing the optical sensor at a level sufficient to visualise individual axonal boutons, and (b) responded to electric stimulation of Schaffer collaterals with the iGluSnFR signal rise. For time-lapse imaging of the iGluSnFR signal (before, during, and after evoked glutamate release), images were collected in frame scan mode to provide fast acquisition rates and a short pixel dwell time. Frame scans were acquired with a pixel dwell time of 0.5 μs, at a nominal resolution of ~ 5–7 pixels per μm (256 × 96). To minimize photodamage, only a single focal section through the region of interest (ROI) containing selected axonal fragments was acquired, at a relatively low laser power (3–6 mW under the objective). The focal plane was regularly adjusted, to account for specimen drift. Time-lapse frame scans of ROIs (containing multiple boutons within the focal plane) were acquired before and up to 60–90 min after the induction of LTP, as detailed below.

The optical signal of the iGluSnFR was expressed as the (*F* (*t*)*- F*_0_)/ *F*_0_ = *ΔF/F*_0_, where *F*(*t*) stands for intensity over time, and *F*_0_ is the baseline intensity averaged over ~ 150 ms prior to the stimulus. To quantify LTP-induced changes in the average optical glutamate signal, we calculated the {*ΔF/F*_0_} value representing the mean *ΔF/F*_0_ signal over the 300 ms interval from the first stimulus onset.

### Electrophysiology *ex vivo:* LTP induction

Glutamate release was evoked by stimulation of the bulk of Schaffer collaterals, using a concentric bipolar electrode (100 μs, 20–200 μA; corresponding to approximately one third of the saturating reponse) placed in the *S. radiatum*. Evoked field excitatory postsynaptic potentials (fEPSPs) were monitored with an extracellular recording pipette positioned in *S. radiatum* > 200 μm away from the stimulating electrode. The recording electrode has a resistance of 1.5–2 MΩ when filled with a Ringer solution. fEPSPs were recorded using a Multipatch 700B amplifier controlled by the pClamp 10.2 software (Molecular Devices, USA).

Synaptic responses were evoked by a brief burst of stimuli consisting of five pulses applied at 20 Hz, 50 ms apart. Basal synaptic transmission was monitored for 10 to 20 min (every 30 s to 1 min, ~ 0.03 Hz) before implementing a high-frequency stimulation (HFS) protocol for the induction of LTP. The HFS protocol contained of three trains of stimuli (100 pulses at 100 Hz), applied in a 60-s interval. GABA receptors were blocked with 100 μM picrotoxin and 3 μM CGP-52432 (in bath). The fEPSP slope was typically monitored for at least an hour (up to 2 h) post-HFS, using the same stimulation protocol (five pulses at 20 Hz).

### Statistical analyses

All data are presented as mean ± standard error of the mean (SEM), with *n* referring to the number of slices analysed. For the statistical difference between baseline and two time points after LTP conditions, paired-sample comparison (paired-sample *t*-test) was performed for the {*ΔF/F*_0_} values, as described.

## Data Availability

The datasets obtained and/or analysed during the current study are available from the corresponding author on reasonable request.
