# Correction to: Optical monitoring of glutamate release at multiple synapses in situ detects changes following LTP induction

**DOI:** 10.1186/s13041-020-00590-9

**Published:** 2020-03-25

**Authors:** Olga Kopach, Kaiyu Zheng, Dmitri A. Rusakov

**Affiliations:** Queen Square Institute of Neurology, University College London, Queen Square, London, WC1N 3BG UK

**Correction to: Mol Brain**

**https://doi.org/10.1186/s13041-020-00572-x**

In the original publication of this article [1], text has been introduced erroneously to Figs. 4a and 5d due to a typesetting mistake. In this Correction the incorrect and correct version of these Figures are shown. The original publication of this article has been corrected.

The publisher apologises to the readers and authors for the inconvenience.

Originally Figs. 4 and 5 were published as:

**Fig. 4:**
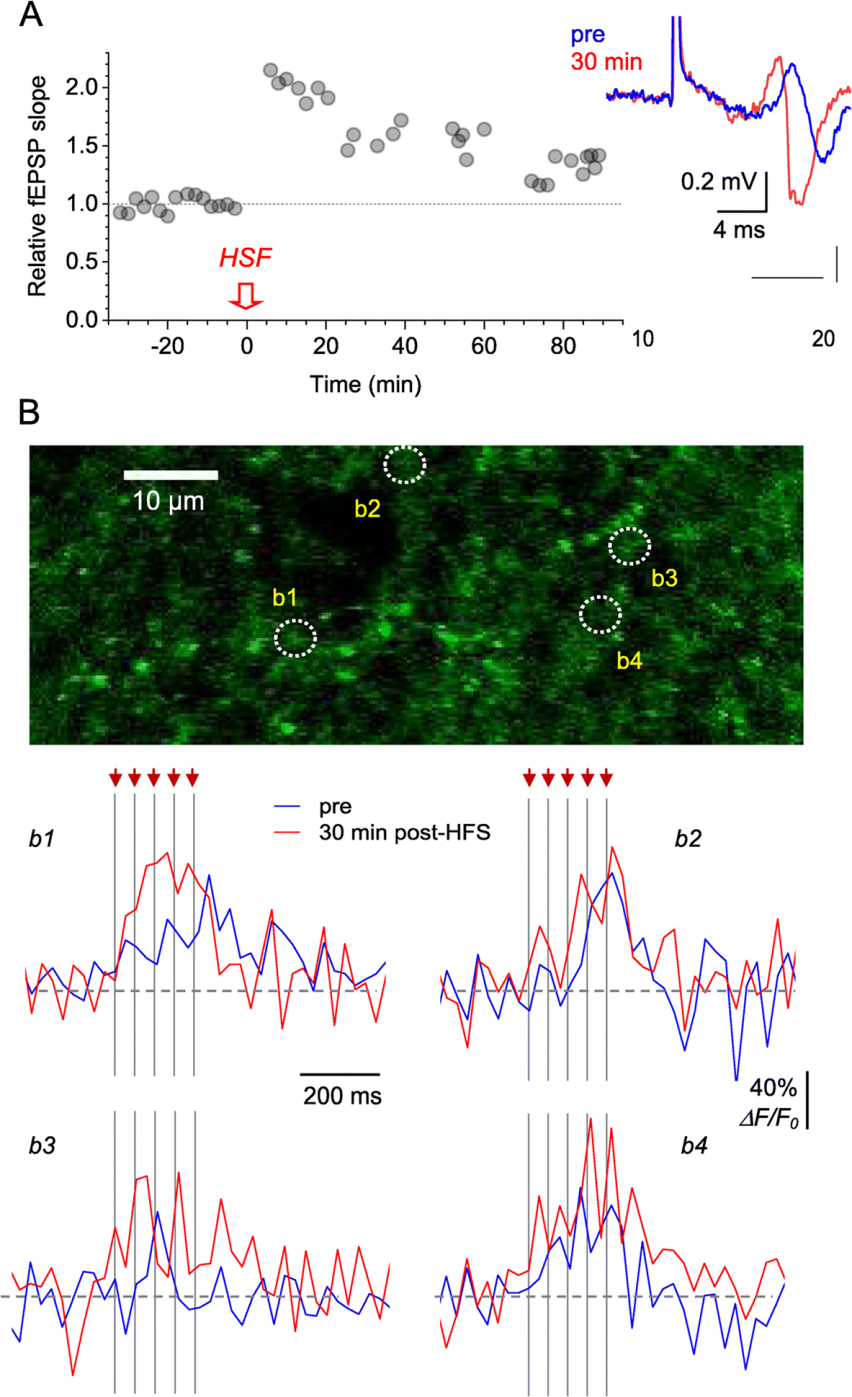
Optical glutamate signal at individual axonal boutons during LTP induction. **a** Characteristic time course of the fEPSP slope recorded in *S. radiatum* following LTP induction by high frequency stimulation (HFS, one-slice example). Traces, the corresponding fEPSP examples in baseline conditions (blue) and 30 min after LTP induction (red). **b** Image, ROI in *S. radiatum* (iGluSnFR.WPRE.SV40 channel) showing 4 axonal boutons, b1-b4, designated for glutamate release monitoring. Traces, iGluSnFR *ΔF/F*_0_ signal recorded from boutons b1-b4 before (blue) and ~ 30 min after (red) LTP induction. Traces are single-trial examples; arrows and dotted lines, afferent stimulus timestamps

**Fig. 5:**
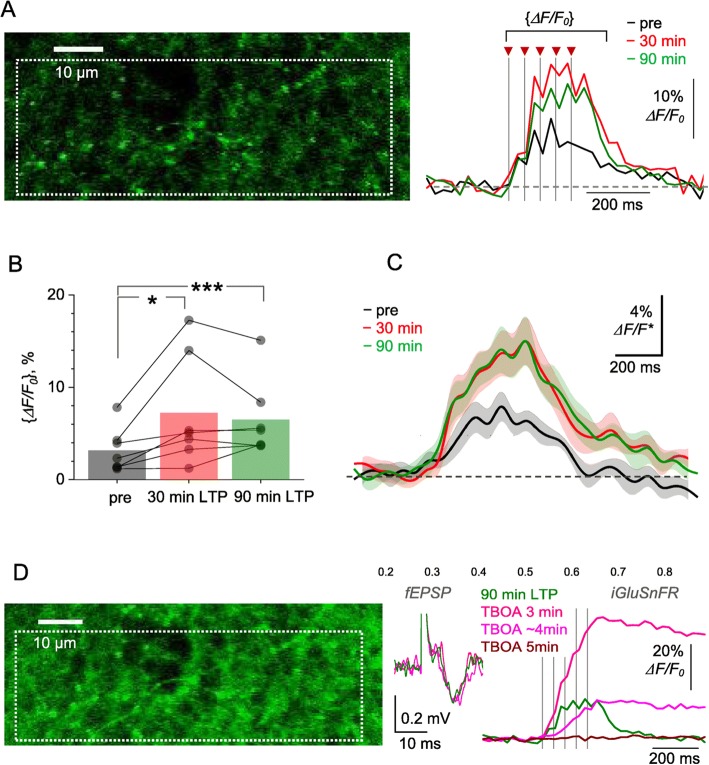
LTP induction at CA3-CA1 synapses boosts optical glutamate signal in the *S. radiatum* neuropil. **a** Image, axon fragment in *S. radiatum* showing the area with multiple axonal boutons (dotted rectangle, iGluSnFR.WPRE.SV40 channel) for the analysis of average iGluSnFR *ΔF/F*_0_ signal (right traces), as shown before (pre), ~ 30 min after (red), and 90 min after HFS. One-slice example; traces, single-trial examples; arrows and dotted lines, afferent stimulus timestamps. Averaging interval for calculating {*ΔF/F*_0_} values is shown. **b** ROI-average iGluSnFR {*ΔF/F*_0_} values in baseline conditions (pre), and at 30 min and 90 min after LTP induction, as indicated. Connected dots, individual slice data; bars, average values (*n* = 7). *p < 0.04; ***p < 0.005. **c** Average iGluSnFR *ΔF/F*_*0*_ signal traces (line ± shaded area, mean ± SEM, n = 7) normalised to their {*ΔF/F*_0_} value in baseline conditions, in each individual preparation, and rescaled to illustrate the ‘average *ΔF/F*_0_ traces’ across preparations (ΔF/F*). **d** Experiment as in (**a**) but following the blockade of glutamate transporters with 50 μM TBOA, at 90 min after LTP induction. fEPSP and iGluSnFR traces illustrate single trials recorded at different time points after TBOA application onset, as indicated; one-slice example, notations as in (**a**). Note that no *ΔF/F*_*0*_ signal (red) may reflect saturation of the baseline fluorescence *F*_*0*_

The correct version of Figs. 4 and 5:

**Fig. 4:**
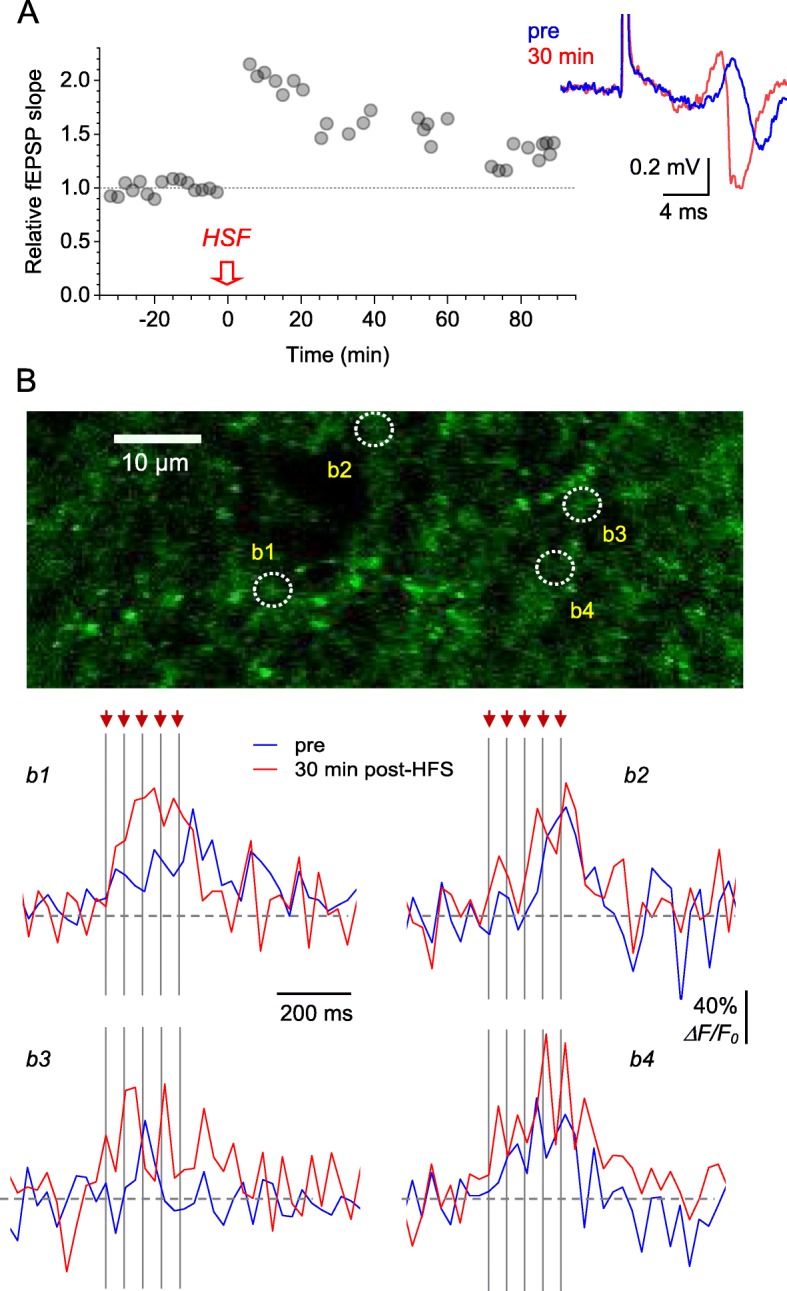
Optical glutamate signal at individual axonal boutons during LTP induction. **a** Characteristic time course of the fEPSP slope recorded in *S. radiatum* following LTP induction by high frequency stimulation (HFS, one-slice example). Traces, the corresponding fEPSP examples in baseline conditions (blue) and 30 min after LTP induction (red). **b** Image, ROI in *S. radiatum* (iGluSnFR.WPRE.SV40 channel) showing 4 axonal boutons, b1-b4, designated for glutamate release monitoring. Traces, iGluSnFR *ΔF/F*_0_ signal recorded from boutons b1-b4 before (blue) and ~ 30 min after (red) LTP induction. Traces are single-trial examples; arrows and dotted lines, afferent stimulus timestamps

**Fig. 5:**
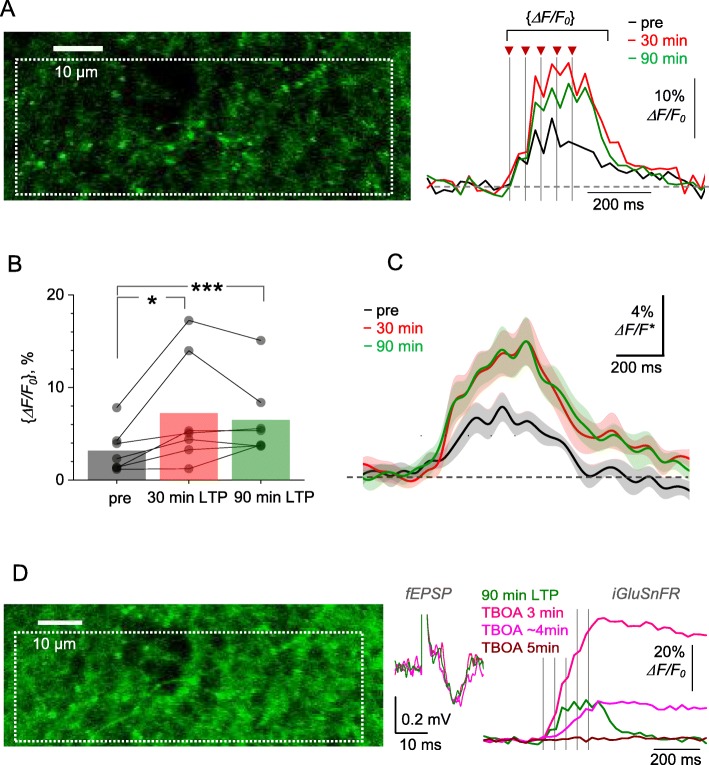
LTP induction at CA3-CA1 synapses boosts optical glutamate signal in the *S. radiatum* neuropil. **a** Image, axon fragment in *S. radiatum* showing the area with multiple axonal boutons (dotted rectangle, iGluSnFR.WPRE.SV40 channel) for the analysis of average iGluSnFR *ΔF/F*_0_ signal (right traces), as shown before (pre), ~ 30 min after (red), and 90 min after HFS. One-slice example; traces, single-trial examples; arrows and dotted lines, afferent stimulus timestamps. Averaging interval for calculating {*ΔF/F*_0_} values is shown. **b** ROI-average iGluSnFR {*ΔF/F*_0_} values in baseline conditions (pre), and at 30 min and 90 min after LTP induction, as indicated. Connected dots, individual slice data; bars, average values (*n* = 7). *p < 0.04; ***p < 0.005. **c** Average iGluSnFR *ΔF/F*_*0*_ signal traces (line ± shaded area, mean ± SEM, n = 7) normalised to their {*ΔF/F*_0_} value in baseline conditions, in each individual preparation, and rescaled to illustrate the ‘average *ΔF/F*_0_ traces’ across preparations (ΔF/F*). **d** Experiment as in (**a**) but following the blockade of glutamate transporters with 50 μM TBOA, at 90 min after LTP induction. fEPSP and iGluSnFR traces illustrate single trials recorded at different time points after TBOA application onset, as indicated; one-slice example, notations as in (**a**). Note that no *ΔF/F*_*0*_ signal (red) may reflect saturation of the baseline fluorescence *F*_*0*_
